# The Role of Iron Regulation in Immunometabolism and Immune-Related Disease

**DOI:** 10.3389/fmolb.2019.00116

**Published:** 2019-11-22

**Authors:** Shane J. F. Cronin, Clifford J. Woolf, Guenter Weiss, Josef M. Penninger

**Affiliations:** ^1^IMBA, Institute of Molecular Biotechnology of the Austrian Academy of Sciences, Vienna, Austria; ^2^Department of Neurobiology, Harvard Medical School, Boston, MA, United States; ^3^FM Kirby Neurobiology Center, Boston Children's Hospital, Boston, MA, United States; ^4^Department of Internal Medicine II (Infectious Diseases, Immunology, Rheumatology and Pneumology), Medical University of Innsbruck, Innsbruck, Austria; ^5^Christian Doppler Laboratory for Iron Metabolism and Anemia Research, Medical University of Innsbruck, Innsbruck, Austria; ^6^Department of Medical Genetics, Life Sciences Institute, University of British Columbia, Vancouver, BC, Canada

**Keywords:** iron, anemia, infection, mitochondria, BH4

## Abstract

Immunometabolism explores how the intracellular metabolic pathways in immune cells can regulate their function under different micro-environmental and (patho-)-physiological conditions (Pearce, 2010; Buck et al., 2015; O'Neill and Pearce, 2016). In the last decade great advances have been made in studying and manipulating metabolic programs in immune cells. Immunometabolism has primarily focused on glycolysis, the TCA cycle and oxidative phosphorylation (OXPHOS) as well as free fatty acid synthesis and oxidation. These pathways are important for providing the energy needs of cell growth, membrane rigidity, cytokine production and proliferation. In this review, we will however, highlight the specific role of iron metabolism at the cellular and organismal level, as well as how the bioavailability of this metal orchestrates complex metabolic programs in immune cell homeostasis and inflammation. We will also discuss how dysregulation of iron metabolism contributes to alterations in the immune system and how these novel insights into iron regulation can be targeted to metabolically manipulate immune cell function under pathophysiological conditions, providing new therapeutic opportunities for autoimmunity and cancer.

## Introduction

Iron is one of the most abundant elements on Earth and essential to almost all organisms. Iron exists in a wide range of oxidation states, −2 to +7. Chemically, the most common and biologically relevant oxidation states of iron are +2 and +3, ferrous (Fe^2+^) and ferric (Fe^3+^) iron. Ferrous (Fe^2+^) iron is more soluble and bioavailable than its ferric (Fe^3+^) form and that the interchangeability of these two ionic forms of iron via oxidation/reduction are essential for the function of many cellular proteins. Levels of iron in the body are strictly controlled through finely tuned complex mechanisms, to prevent the cytotoxicity that is induced by accumulation of this metal and to allow physiologically tolerable iron levels to serve as a critical catalytic component of many proteins and enzymes, called metalloproteins.

Metalloproteins can directly bind iron or use iron-containing complexes such as heme or iron-sulfur (Fe-S) clusters. Such proteins have diverse and essential processes within the cell, including oxygen carrying (hemoglobin), oxygen storage (myoglobin), energy production (cytochrome-C), cellular metabolism (amino acid oxidases, fatty acid desaturases), detoxification (cytochrome P450, catalase), and host defense (myeloperoxidase, nitric oxide synthase, IDO, NAPH oxidase) (Muckenthaler et al., 2017). Although the chemistry of iron will not be discussed here in detail, Fenton/Haber-Weiss chemistry is a very important reaction with widespread effects on biological systems under normal and pathophysiological conditions: ferrous (Fe^2+^) iron reacts with hydrogen peroxide to form the hydroxyl ion (OH^−^), the hydroxyl radical (OH•) and ferric (Fe^3+^) iron (Koskenkorva-Frank et al., 2013). The OH• radical is a non-selective, highly toxic oxidant. As mitochondria produce ATP by oxidative phosphorylation (OXPHOS), reactive oxygen species (ROS) by-products such as superoxide are generated from the electron transport chain (ETC). Superoxide radicals can reduce and liberate Fe^3+^ from ferritin or liberate Fe^2+^ from Fe-S clusters (see below). Biologically-available iron not sequestered is thus a dangerous source of damaging radicals (Breuer et al., 2008). It is important to note that not all free radicals are detrimental and not all antioxidants are beneficial. Normal physiology is a balance between the two: antioxidants maintain levels of ROS that permit them to perform useful biological functions, such as neutrophil-mediated killing of phagocytosed bacteria or enhanced T cell proliferation after TCR stimulation, while minimizing by-stander damage. However, under pathophysiological conditions, such as enhanced mitochondrial stress, this balance gets perturbed to the detriment of the organism.

**Figure 1:**
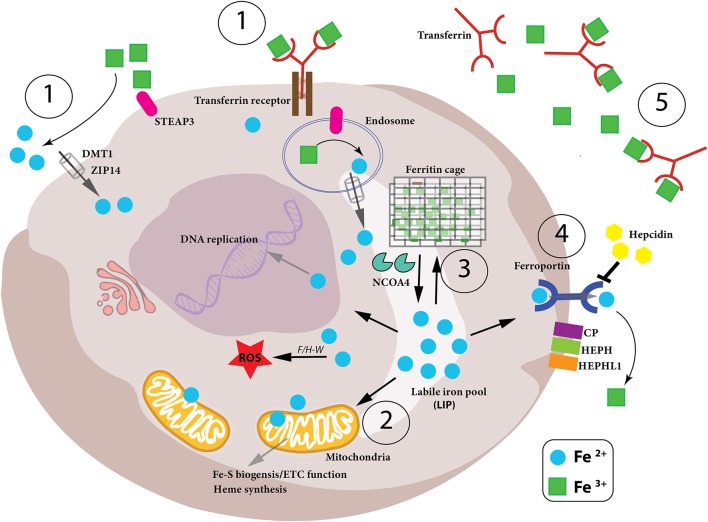
Iron metabolism in the cell. Intracellular iron levels are strictly controlled as too little or too much can be detrimental to the health of the cell. Therefore, (1)-iron uptake, (2)-utilization, (3)-storage and (4)-export need to be managed in a coordinated manner, as well as the conversion between the oxidation states of iron (Fe^2+^ and Fe^3+^) in the cell. (1) Iron-bound transferrin (TF-Fe^3+^) and NTBI (non-transferrin-bound iron) are taken up into the cell by the iron importers DMT1 and ZIP14. STEAP3 is a ferrireductase which reduces Fe^3+^ to Fe^2+^, which can then be imported. (2) Once inside the cell, the bioavailable and more soluble Fe^2+^ is used for various biological processes– DNA replication, ROS production via Fenton/Haber-Weiss (F/H-W) chemistry, mitochondrial bioenergetics, Fe-S and heme biosynthesis, as well as a plethora of proteins which utilize the metal to carry out their functions. (3) Excess Fe^2+^ iron is dangerous due to its role in ROS production. Therefore, it needs to be stored but, at the same time, be readily available for use. This is achieved by a particular arrangement of ferritin proteins designated the “ferritin cage” which stores the more inert, insoluble Fe^3+^ form of iron. When intracellular levels are low, this ferritin cage is signaled for destruction by NCOA4 thus releasing the stored iron. (4) If intracellular iron levels are saturated, then the iron must be exported out of the cell. This is achieved by the iron exporter ferroportin (FPN). Once outside the cell the Fe^2+^ iron is oxidized to Fe^3+^ (via CP, HEPH, HEPHL). (5) Finally, Fe^3+^ iron is then bound to transferrin (Tf-Fe^3+^) and enters the circulation to begin the cycle again. Notably, hepcidin is an iron-controlling hormone produced by the liver. When systemic iron levels are high in the blood, hepcidin is produced and leads to the degradation of FPN on cells thus preventing cellular release of iron into the blood. Conversely, when iron blood levels are low, hepcidin expression is reduced.

Iron is essential for many physiological processes in the body including erythropoiesis, immune function and host defense, as well as essential cellular activities such as DNA replication and repair, mitochondrial function including OXPHOS and enzymatic reactions which require iron as a cofactor. Extensive research by many groups has unveiled the regulatory network governing iron homeostasis in the body and inside the cell, as well as the links between disturbances of iron homeostasis and disease. Iron deficiency is the most common pathology of iron homeostasis, eventually resulting in iron deficiency anemia, the most frequent anemia worldwide (Camaschella, 2015). The second most frequent anemia, anemia of inflammation (also called anemia of chronic disease), largely results from inflammation-driven retention of iron in certain immune cells, resulting in iron-limited erythropoiesis (Weiss et al., 2019). This latter pathology reflects the complex regulatory interactions between iron and the immune system, which emerged evolutionary from a strategy of the organism to withhold nutrient iron from invading pathogens, a defense mechanism known as nutritional immunity. Accordingly, iron trafficking is controlled by cytokines and acute phase proteins, whereas the metal itself promotes lymphocyte and macrophage differentiation, anti-microbial immune effector function, and immune cell metabolism, as we will discuss later (Ganz and Nemeth, 2015; Soares and Weiss, 2015). Thus, imbalances in iron homeostasis are prevalent in infections, cancer as well as autoimmunity, and its pathophysiological or therapeutic modulation impacts on the outcome of such diseases. Evolution reveals its mastery in the way the body and immune cells strike a balance between iron supply and demand with pathways tightly regulating iron levels extra- and intra-cellularly, from its uptake, use, storage, and export, collectively referred to as the iron cycle (Figure 1, Table 1). In the next sections we will describe the various stages of the iron cycle and give an overview of how iron levels are monitored and regulated inside the cell. This cellular regulation of iron is applicable to practically every cell in the body, including all immune cells.

**Table 1:** The major players of the Iron Cycle.

**Process**	**Protein**	**Location**	**Function**
Iron intestinal uptake	DcytB DMT1	Gut lumen > enterocyte Gut lumen > enterocyte	Ferrireductase (reduces Fe^3+^ to Fe^2+^) iron transporter of Fe^2+^
	Unidentified HO1 HO2	Gut lumen > enterocyte inside enterocyte	Heme-conjugated iron Breaks down the heme to produce free Fe2+
	PCBP2	Inside enterocyte	Chaperones Fe^2+^ to basolateral side of enterocyte
Release of dietary iron to circulation	FPN Hephaestin	Enterocyte > circulation	Fe^2+^ exporter from enterocyte Ferroxidase (oxidizes Fe^2+^ to Fe^3+^)
In the circulation	TF NTBI	In the blood In the blood	TF binds and transports Fe^3+^ (TF-Fe^3+^ complex) Non-transferrin bound iron
Cellular iron uptake	TFR1 Low pH STEAP3 DMT1	Cell surface Endosome Endosome Endosome > cytosol	Binds and endocytoses TF-Fe^3+^ Release of Fe^3+^ from TF-Fe^3+^ (TFR1 recycled to surface) Ferrireductase (reduces Fe^3+^ to Fe^2+^) Iron transporter of Fe^2+^
	ZIP14 DMT1	Cell surface > cytosol Cell surface > cytosol	Binds and uptakes NTBI into cell
Intracellular iron storage/release	FTH1 FTL1	Cytosol/mitochondria	Components of “ferritin cage”
	NCOA4	Cytosol	Targets ferritin for autosomal degradation to release iron
Iron cellular export	FPN	Cytosol > circulation	Fe^2+^ exporter from the cell
	CP HEPH HEPHL1	Outer cell surface	Ferroxidase (oxidizes Fe^2+^ to Fe^3+^)

*This table depicts the various stages of the iron cycle, the proteins involved at each stage as well as their function, and the location-of-action of these proteins*.

## Iron Cycle—Dietary Uptake of Iron

We absorb 1–2 mg of iron daily through the duodenal intestinal epithelium. Intestinal enterocytes are responsible for dietary iron uptake in the form of heme iron and ionic iron. When systemic iron levels in the blood stream are low, expression of DMT1 (divalent metal transporter 1, also called NRAMP2 as well as SLC11A2) and DcytB (duodenal membrane associated cytochrome-b) ferrireductase increases on the surface of intestinal enterocytes on the luminal side enabling the reduction of Fe^3+^ into its ferrous form (Fe^2+^) which can then be taken up by DMT1 (McKie et al., 2001). It has also been demonstrated that heme-conjugated iron is absorbed by enterocytes in the duodenum although this heme importer has not yet been characterized (Gräsbeck et al., 1979). Once internalized heme is degraded by HO-1 and HO-2 (heme-oxygenase 1/2) to produce free Fe^2+^ (Maines, 1988). Inside the enterocyte, Fe^2+^ iron is next chaperoned to the basolateral surface of the cell by PCBP2 (poly-(rC)-binding protein 2) where the iron is then released into the circulation for systemic use (Yanatori et al., 2016). This latter step is controlled by the iron exporter, ferroportin (FPN, also called SLC40A1) followed by extracellular oxidation to ferric iron by the copper enzyme hephaestin (HEPH) (Vulpe et al., 1999; Muckenthaler et al., 2017). Ferric iron in the circulation is then bound by transferrin (Tf) and carried to cells and tissues (Muckenthaler et al., 2017). However, the major source of iron for systemic needs, such as red blood cell (RBC) maturation, are macrophages, which ingest senescent erythrocytes as well as haptoglobin-bound free hemoglobin and heme/Fe complexes released from lysed RBCs (Burger et al., 2012; Chow et al., 2013). The iron taken up by macrophages is re-utilized by HO-1 and then shuttled to the circulation via FPN. This macrophage-dependent process accounts for ~90–95% of the daily needs of iron, giving a clear example of the close link between iron homeostasis and immunity (Crichton and Ward, 1998).

## Iron Cycle—Cellular Uptake of Iron

Transferrin (Tf) is a glycoprotein containing two high affinity binding sites for ferric iron (Fe^3+^) (Aisen et al., 1978), which captures Fe^3+^ in the circulation (Figure 1). This diferric Tf conjugate (Tf-Fe^3+^) not only prevents free, non-transferrin bound iron (NTBI), from engaging in Fenton chemistry to produce dangerous hydroxyl radicals, but also deprives invading pathogens of free iron to block their expansion and proliferation (Barber and Elde, 2014). Tf-Fe^3+^ binds to the high-affinity transferrin receptor (TFR1, CD71) on the surface of cells, and this complex is subsequently endocytosed (Harding et al., 1983). Within the acidic environment of the early endosome, the ferric iron is released from the Tf-TFR1 complex which itself is recycled back to the membrane where TFR1 is reinserted and Tf is released back to the circulation. The freed ferric iron is then reduced to its bio-active ferrous form, Fe^2+^ by the ferrireductase STEAP3 (six-transmembrane epithelial antigen of prostate 3) and shuttled into the cytosol from the endosome by DMT1 (Fleming et al., 1997; Gunshin et al., 1997; Ohgami et al., 2005; Figure 1). The importance of iron uptake into cells is clearly evident from genetic mouse studies where ablation of the *Tfr1* gene results in detrimental pathologies including cardiomyopathy, muscle atrophy, dopaminergic neurodegeneration, and severe anemia due to reduced erythrocyte development (Levy et al., 1999; Barrientos et al., 2015; Xu et al., 2015; Matak et al., 2016). Of note, humans mutations in the *TFR1* gene have been associated with severe combined immunodeficiency (Jabara et al., 2015). These reports demonstrate how certain cell types rely more heavily on TFR1-mediated iron uptake while other cell types have adapted other mechanisms to import iron into their cells. Notably, as we discuss later, iron not readily used for metabolic purposes is stored by the protein ferritin and ferritin-conjugated iron released from various cells is taken up by Scara5 (Scavenger receptor class A member 5) or TIM-2 (T Cell Immunoglobulin And Mucin Domain Containing 2) receptors (Chen et al., 2005). Furthermore, free heme and hemoglobin released during red blood cell (RBC) lysis are bound in the circulation by hemopexin and haptoglobin, respectively, and these iron-containing complexes are then taken up by cells expressing the CD91 and CD163 receptors (Nairz et al., 2017). In the circulation there is also non-transferrin bound iron (NTBI) which can be taken up into the cell by ZIP- (ZRT/IRT-like protein)-14 or DMT1 (Ludwiczek et al., 2003; Liuzzi et al., 2006; Pinilla-Tenas et al., 2011; Figure 1); the ferrireductase activity of the prion protein (PRNP) as well as cellular reductants released by the cell (such as ascorbate) reduces Fe^3+^ iron to Fe^2+^ iron to facilitate this transport (Lane and Lawen, 2008; Tripathi et al., 2015). After uptake and reduction, ferrous Fe^2+^ iron enters the cytosol where it is collectively referred to as the “labile iron pool (LIP).” It is from this Fe^2+^-laden pool, that iron homeostasis is strictly regulated according to the needs of the cell, whether iron is utilized, stored for future use or exported out of the cell to prevent iron overload and oxidative damage (Figure 1).

## Iron Cycle—Mitochondrial Utilization of Iron

Most of the LIP is trafficked to mitochondria, the energy producing batteries of the cell. The mitoferrin transporters (Mitoferrin1 and Mitoferrin2) are responsible for the mitochondrial import of iron (Shaw et al., 2006; Troadec et al., 2011; Chung et al., 2014). Once inside the organelle the iron is incorporated into heme and iron-sulfur (Fe-S) clusters by frataxin and GLRX5 (Glutaredoxin-related protein 5) (Lill, 2009; Braymer and Lill, 2017). Frataxin has been proposed to provide the iron while GLRX5 acts not only as a scaffolding protein but may also facilitate the transfer of Fe-S clusters to target proteins (Yoon and Cowan, 2003; Ye et al., 2010). Heme is essentially a conjugate complex of iron and porphyrin IX. These heme complexes are then shuttled out of the mitochondria to the cytosol by the Feline Leukemia Virus Subgroup Receptor 1 (FLVCR1) (Tailor et al., 1999) where hemoglobin in erythrocytes, or other proteins known as hemoproteins, incorporate the heme complex to confer functionality; the heme iron component acts as a platform to receive or provide electrons during redox chemistry while for transportation of oxygen in hemoglobin or myoglobin, the gas binds to the heme iron (Milani et al., 2005).

Mitochondrial iron is also required for the synthesis of Fe-S clusters and indeed their biogenesis is thought to be one of the most important functions of the mitochondria—even though some Fe-S assembly can occur in the cytosol and nucleus (Tong, 2000; Tong et al., 2003; Tong and Rouault, 2006). Fe-S clusters are ubiquitous, inorganic co-factors that contribute to a wide range of cellular pathways from genome integrity and gene regulation to energy production and immune responses, and are required for numerous biological functions, particularly enzymatic activity (Beinert, 2000; Johnson et al., 2005; Lee et al., 2009; Fuss et al., 2015). Their assembly is highly conserved throughout evolution from yeast and bacteria to human. The sulfur is provided by NFS1, a cysteine desulfurase which removes a sulfur moiety from L-cysteine (Biederbick et al., 2006). The Fe source of the cluster is not yet known, although frataxin (FXN) has been identified as an important iron regulator of Fe-S biogenesis (Colin et al., 2013). Iron–sulfur clusters are found in a variety of metalloproteins, such as the ferredoxins, NADH dehydrogenase, and hydrogenases. Fe-S clusters are also involved in electron transfer, made possible because iron can exist stably in either the +2 (ferrous) or +3 (ferric) oxidation states. Complexes I, II, and III of the electron transfer complex (ETC) of OXPHOS in mitochondria require numerous Fe-S clusters to function efficiently (Figure 1). It is therefore not surprising that mutations or dysfunctions of Fe-S cluster biogenesis have been linked to multiple inherited disorders such as Friedreich's ataxia (caused by a disruptive guanine-adenine-adenine repeat in the first intron of FXN) (Campuzano et al., 1996), infantile complex II/III deficiency syndrome (a lethal autosomal recessive disease caused by a point mutation in NFS1) (Farhan et al., 2014) and multiple mitochondrial dysfunctions syndromes (MMDS1-3, due to mutations in genes involved in Fe-S biogenesis and characterized by severely reduced mitochondrial function) (Wachnowsky et al., 2017). All these pathological conditions affect multiple organs including the nervous system, muscles and the immune system highlighting the importance of iron regulation in mitochondria. Indeed, a recent article shows the evolutionary conservation for the need of dietary iron for proper mitochondrial function. *Caenorhabditis elegans* fed on a mutant strain of *Escherichia coli*, which exhibits reduced iron uptake, lead to diminished iron uptake by the worms resulting in significantly impaired mitochondrial ETC function, enhanced ROS production and developmental defects (Zhang et al., 2019).

## Iron Cycle—Intracellular Storage of Iron

Iron from the LIP which is not used for metabolic processes is stored in the cytosol in the “ferritin nano-cage,” a cytosolic heteropolymer composed of 24 subunits of heavy (FTH1) and light (FTL1) ferritin chains (Lawson et al., 1991; Figure 1). The multi-subunit caged ferritin can withstand high temperatures and a wide range of pH levels for limited periods as is the necessity to prevent free Fe^2+^ iron to engage in uncontrolled ROS production. The ferritin nanocage stores iron in an insoluble non-toxic state (Fe^3+^) in cells while keeping it bioavailable by converting it to its soluble form (Fe^2+^) when required. The iron in the central cavity is maintained as small crystalline particles in its ferric Fe^3+^ form due to the ferroxidase activity of FTH1 (Hentze et al., 1986; Yang and Chasteen, 1999). There also exists a mitochondrial form of ferritin (Levi et al., 2001) which may both protect mitochondria against iron-mediated toxicity and provide a quick and efficient source of iron in this organelle rather than relying solely on the cytosolic ferritin. When intracellular levels of iron are depleted, the ferritin-sequestered iron can be made available through ferritin degradation. Lows levels of iron signal to NCOA4 (nuclear receptor coactivator 4) to target the ferritin complex for autolysosome degradation, a process termed “ferritinophagy” (Santana-Codina and Mancias, 2018).

NCOA4 is a key regulator of ferritinophagy and intracellular iron levels—NCOA4-null mice are unable to undergo ferritinophagy and this increased retention of iron within ferritin complexes results in reduced iron export from cells and ultimately in iron-deficient anemia (Bellelli et al., 2014, 2016). Conversely, in iron-loaded cells the turnover of NCOA4 is greatly enhanced to block ferritin degradation and thus increase iron storage (Mancias et al., 2014, 2015; Bellelli et al., 2016; Santana-Codina and Mancias, 2018). Ferritin levels are also regulated by inflammation and oxidative stress. NF-kB, an important integrator transcription factor downstream from multiple inflammatory signals, as well as NRF2 (nuclear factor erythroid 2-related factor-2), the main stress-responsive transcription factor, both increase ferritin transcription (Miller et al., 1991; Pham et al., 2004). Furthermore, proinflammatory cytokines, such as interleukin- (IL)-6 and IL-1, stimulate ferritin translation (Rogers, 1996). These regulatory pathways ensure that any free Fe^2+^ iron is stored away, inaccessible for bacteria or other invading pathogens as well as from producing dangerous radicals through Fenton/Haber-Weiss chemistry.

## Iron Cycle—Iron Export From the Cell

When iron levels within a cell are at levels where it is being effectively used and stored, then any excess iron will be exported from the cell to prevent intracellular iron overload. The key iron exporter is ferroportin (FPN, also called SLC40A1) which exports Fe^2+^ iron (McKie et al., 2000). At the outer cell membrane iron is oxidized to its ferric (Fe^3+^) form by three iron oxidases each displaying distinct expression patterns—ceruloplasmin (CP) (Miller and Cohen, 2001), hephaestin (HEPH) (Vulpe et al., 1999), and zyklopen (HEPHL1) (Chen et al., 2010; Figure 1). The importance of these oxidases for successful iron export into the circulation has been demonstrated with gene-ablation murine models as well as identification of patients deficient in *CP* and *HEPH*, presenting with iron-deficient anemia (Yoshida et al., 1995; Hahn et al., 2004). Although FPN is ubiquitously expressed by most cells in the body, it is abundantly expressed by those cell types which contribute extensively to plasma iron levels; these cells include intestinal enterocytes (to traffic dietary iron into the circulation), macrophages (to re-circulate iron originating from phagocytosis of damaged or aged red blood cells) as well as hepatocytes.

The major need of iron in the body is for erythropoiesis, the production of oxygen-transporting RBCs; however, iron is also needed in muscle cells where it is incorporated into myoglobin. As there exists no effective excretion mechanism for iron surplus, iron is stored in parenchymal organs, mainly the liver. Hepatic iron deposits can range from 300 mg to 1 g, but can reach up to 25–30 g in patients suffering from genetic iron overload, a condition called hereditary hemochromatosis (HH) (Janssen and Swinkels, 2009; Anderson and Shah, 2013; Procaccini and Kowdley, 2017). Different forms of hemochromatosis have been described which all have a reduced production of the hormone, hepcidin (Pietrangelo, 2007; Weiss, 2010; Figure 1). The expression of hepcidin in the liver is transcriptionally controlled by bone morphogenic proteins (BMP)- SMAD mediated signaling cascades which are regulated by different factors such as iron deficiency, iron overload, hypoxia, inflammation, infection, and hormones (Girelli et al., 2016; Silvestri et al., 2019). Hepcidin then enters the circulation and binds to the extracellular part of FPN targeting it for degradation, thus preventing iron from being exported into the circulation and instead being sequestered in the cell (Nemeth et al., 2004). This results in an overall reduction of iron in the bloodstream. Hepcidin overexpression leads to low plasma iron levels (hypoferremia) and anemia (Weinstein et al., 2002; Roy et al., 2007; Theurl et al., 2009; Altamura et al., 2014). Conversely, individuals with insufficient hepcidin production, suffer from iron-overload in the blood, and hepatitis (Pietrangelo, 2004). The latter is due to the fact, that several co-factors expressed on the hepatocyte cell membrane such as HFE, hemojuvelin (HJV) or TfR2 impact on hepcidin expression via the BMP-SMAD pathway (Babitt et al., 2006). Mutations in these genes lead to impaired hepcidin formation and ultimately to increased iron absorption which is then stored in the liver causing organ damage over time. Hepcidin production is also counter-regulated by developing red blood cells in the marrow. Erythroferrone (ERFE) is produced by erythropoietin (EPO)-stimulation of erythroid progenitors and blocks hepcidin synthesis in the hepatocytes (Kautz et al., 2014). Indeed, EPO itself can reduce hepcidin production via inhibition of the BMP-SMAD pathway (Gammella et al., 2015) and several proteins induced by hypoxia such as hypoxia inducible factor-1 (HIF-1) or platelet derived growth factor BB (PDGF-BB) reduce hepcidin expression, thereby enhancing iron delivery for erythropoiesis (Peyssonnaux et al., 2007; Sonnweber et al., 2014).

**Figure 2:**
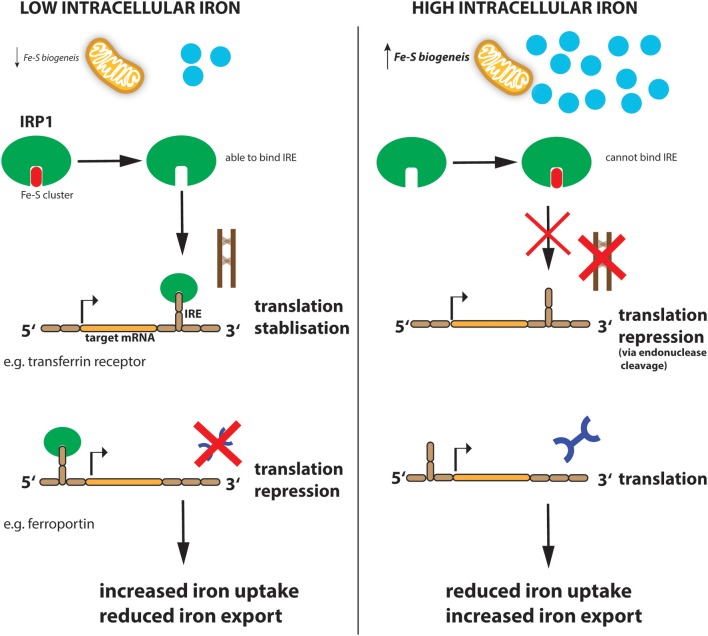
Regulation of iron metabolism by IRP1. When cellular iron levels are low, Fe-S biogenesis in the mitochondria is reduced and IRP1 loses its Fe-S cluster component. This allows IRP1 to now bind to the IRE sequence in target mRNAs. IRP1 binds to the IREs at either the 3′ (for example transferrin mRNA) or 5′ (for example ferroportin mRNA) untranslated region (UTR) of the targeted mRNA. Binding to the 5′ UTR blocks translation while binding to the 3′ UTR stabilizes the mRNA against endonuclease cleavage. Thus, when iron levels are low (left panel), transferrin protein is enhanced while ferroportin protein is reduced resulting in increased iron import and reduced export, to ultimately increase intracellular iron levels. When iron levels are high (right panel), this leads to reduced iron uptake and increased export.

Similar to systemic iron levels in the bloodstream, the intracellular iron levels are tightly controlled. Imbalanced iron levels in the cell can lead to dysfunctional mitochondria, increased oxidative damage and increased toxicity (Richardson et al., 2010; Volani et al., 2017). It is the job of the iron-regulating proteins (IRPs), IRP1 and IRP2, in the cell to react to cytosolic iron concentrations by controlling the translation of proteins involved in iron uptake, use, storage and export. This mechanism is important not only for normal iron homeostasis but also as a host defense in macrophages infected with intracellular bacteria such as *Salmonella* (Nairz et al., 2015). IRPs accomplish this by binding to specific, non-coding sequences called iron-responsive elements (IREs) in the mRNA of target genes (Recalcati et al., 2010b; Figure 2). IREs are 30-nucleotide long RNA motifs that form special stem-loop structures. IREs occur in either the 3′-UTR (untranslated region) or 5′-UTR of a respective mRNA (Figure 2). IRP1 in its non-IRE binding conformation acts as a cytoplasmic aconitase which converts citrate to isocitrate in the cytoplasm, allowing the cell to balance the amount of NADPH (generated from isocitrate by isocitrate dehydrogenase), with the amount of acetyl-CoA (generated from citrate by citrate lyase) (Wilkinson and Pantopoulos, 2014). Fatty acid synthesis requires both these products, NADPH and acetyl-CoA, as do other metabolic processes. However, IRP1 only has aconitase catalytic activity and performs these functions when intracellular iron concentrations are sufficient in the cell. When iron concentrations are low, IRP1 loses it Fe-S cluster and, therefore, its catalytic abilities but instead can now bind to IREs in the 5′ UTR of mRNAs such as ferritin-H and -L subunits as well as the rate-limiting enzyme of heme biogenesis, ALAS (aminolevulinic acid synthase) which results in inhibition of their translation and thus reduced storage and utilization of the metal (Figure 2). However, IRP1 binding to the 3′ UTR increases the stability of the respective target mRNAs such as TFR and DMT1, thereby promoting their expression and subsequently enhancing iron uptake into the cell to replenish intracellular iron levels. When iron levels normalize again, IRP1 regains its Fe-S cluster and aconitase activity. This results in increased ferritin translation and decreased TFR expression, resulting in reduced Fe uptake (Li et al., 2004; Rouault, 2006; Leipuviene and Theil, 2007; Sanchez et al., 2011; Figure 2). This clever regulatory mechanism of IRP1 also links iron levels intimately to intracellular metabolic programs via its aconitase activity and levels of the key metabolites, NADPH and acetyl-CoA.

IRP2 is similar to IRP1 but does not have aconitase activity and is therefore regulated by a different mechanism. Under iron-loaded conditions, IRP2 interacts with FBXL5 (F-box and leucine-rich repeat protein 5) which promotes IRP2 ubiquitination via the SKP1-CUL1-RBX1 E3 ubiquitin ligase complex and subsequent degradation (Salahudeen et al., 2009; Vashisht et al., 2009). However, when iron levels are low, FBXL5 is itself degraded. This is due to a Fe-binding N-terminal hemerythrin-like (Hr) iron-sensing domain in FBXL5. The Hr domain undergoes a conformational change when iron is not present—this structural change leads to FBXL5 polyubiquitination and degradation resulting in IRP2 accumulation (Thompson et al., 2012). FBXL5 stability is also affected by oxygen levels. Under low oxygen hypoxic conditions, an allosteric-induced stabilizing interaction between FBXL5 and the cytoplasmic Fe-S cluster biogenesis complex CIA is disrupted leading to enhanced FBXL5 instability and increased IRP2 levels, highlighting again the extensive crosstalk between iron regulation and other important processes such as oxygen sensing (Mayank et al., 2019). Indeed, IRP2 deficiency switches cellular metabolic pathways from oxidative phosphorylation (OXPHOS) to aerobic glycolysis (Li et al., 2019) through induction of hypoxia-inducible factors (HIF)-1α and−2α which enhances glycolytic pathway proteins and, at the same time, blocks mitochondrial Fe-S cluster biogenesis and OXPHOS. This is further supported by the fact that iron supplementation to cells, or dietary iron overload in mice, affects citric acid cycle activity by modulating mitochondrial aconitase translation (Kim et al., 1996), which also bears an IRE within its 5′ UTR, and further highlights the interconnected nature of the cellular metabolome, especially mitochondrial function, oxidative phosphorylation, and cellular iron consumption (Oexle et al., 1999; Volani et al., 2018).

The iron cycle is present in almost all cells of the body, and so the regulatory networks described above are also essential to the cells of the immune system. The physiological importance of iron regulation is seen when disturbances of iron metabolism affect immunity and, conversely, how activation of the immune system lead to alterations in iron balance. Both iron deficiency and iron excess can influence both the innate and adaptive arms of the immune system.

## Immune Pathologies Associated With Iron Dysregulation

**Figure 3:**
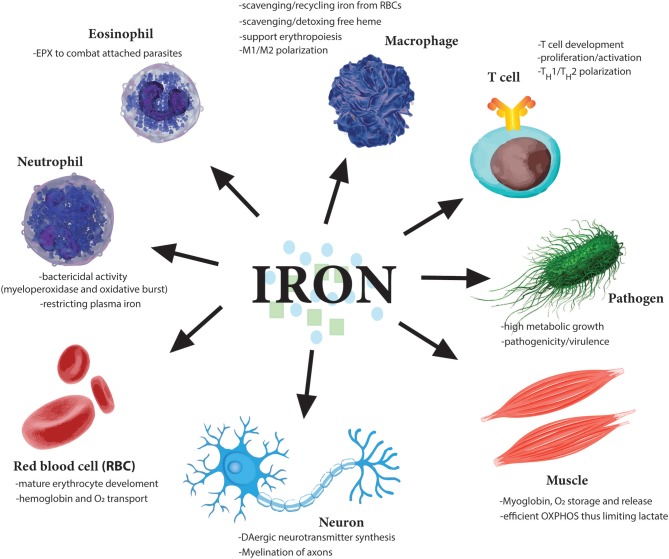
Essential role of iron for various cell types. Iron is needed by many different cell types to perform very distinct functions from oxygen carrying abilities of red blood cells (RBCs) to oxygen storage in muscle cells. Moreover, iron dysregulation has been observed in the pathogenesis of many diseases such as the neurodegenerative diseases Alzheimer's and Parkinson's. Importantly various immune cells regulate iron metabolism to induce their various effector functions. Notably, as iron is so essential for cell division, one of the earliest tasks of invading pathogens is to capture host iron in the circulation to aid their own growth and expansion. Therefore, restricting free iron is a first line of defense against invading pathogens.

Invading pathogens thrive on free iron in the bloodstream to proliferate and advance their attack (Cassat and Skaar, 2013; Figure 3). Hepcidin is an important gatekeeper which promotes intracellular iron retention thus restricting its availability for pathogens in the circulation. However, iron trafficking during infections is differently regulated, depending on the nature and cellular localization, intra- vs. extra-cellular, of the pathogen (Drakesmith and Prentice, 2012; Soares and Weiss, 2015). The importance of “nutritional immunity” as it pertains to iron is exemplified by the increased susceptibility to infection of individuals with iron overload due to thalassemia or primary hemochromatosis, two common genetic diseases in humans (Nairz et al., 2014; Spottiswoode et al., 2014; Arezes et al., 2015). On the other hand, mild iron deficiency is protective against malaria-causing *Plasmodium falciparum* infection (Mabeza et al., 1999; Gwamaka et al., 2012).

Certain intracellular microbes have developed specialized techniques to hijack immune cells' iron withholding network to enhance their own survival by acquiring the metal from the environment. For example, *Mycobacterium tuberculosis* and *Salmonella enterica* reduce FPN expression and iron export allowing more intracellular iron for the bacteria to thrive (Leon-Sicairos et al., 2015). *Neisseria gonorrhoeae* expresses proteins on their surface which recognize human transferrin-Fe (Tf-Fe^3+^) complexes in the bloodstream leading to iron uptake and transport into the bacterial cytoplasm (Ratledge and Dover, 2000). *Vibrio cholerae*, a gram-negative bacterium, is classified as “siderophilic” because its pathogenicity is enhanced by excess iron. Such bacteria can acquire iron directly from Tf-Fe^3+^ complexes in the bloodstream. In healthy individuals Tf-Fe^3+^ concentrations are in the range of 20% but under pathological conditions, as in the case of individuals with HH, it can reach 100% for which *V. cholerae* shows enhanced pathogenicity. A further example is that of *Vibrio vulnificus* which causes sepsis with high mortality rates in individuals with HH and other iron-overloading conditions while healthy individuals are relatively unaffected (Horseman and Surani, 2011). Many bacteria also employ high-affinity iron acquisition pathways to sequester host iron (Skaar and Raffatellu, 2015). Siderophores are low molecular weight iron-binding complexes that are secreted by bacteria to compete with transferrin for iron sequestration (Williams, 1988). However, such pathogenic strategies can be counteracted by the host immune system. For example, lipocalin-2 or neutrophil gelatinase-associated lipocalin (NGAL), a protein secreted by neutrophils, macrophages and epithelia in response to infection binds to siderophores, such as enterobactin, and prevents bacterial iron acquisition by this route. Mice lacking lipocalin-2 exhibit increased mortality from infection with siderophore-expressing bacteria, such as *E. coli*, demonstrating the anti-microbial relevance of this bacterial iron withholding system (Flo et al., 2004; Nairz et al., 2015). However, pathogens are constantly evolving and adapting to host responses and have created modified siderophores, such as the heavily glycoslyated siderophore, salmochelin, secreted by *Salmonella typhimurium*, which is not recognized and targeted by lipocalin-2 thus making these bacteria unresponsive to this host defense strategy (Hantke et al., 2003).

A serious consequence of any infection, or indeed inflammation, is the development of anemia of inflammation (AI) also known as anemia of chronic disease (ACD) (Theurl et al., 2009). The increase of hepcidin within hours of an infection or inflammatory insult, results in a dramatic reduction of plasma iron levels. However, prolonged activation of this defense mechanism can eventually restrict the availability of iron not only to the invading pathogens, but also to developing erythrocytes in the bone marrow, leading to the development of AI (Kim et al., 2014; Weiss et al., 2019). Therefore, the availability and restriction of systemic iron during times of infection and inflammation represents an essential defense mechanism against pathogens but under pathophysiological conditions can lead to anemia and further severe pathologies resulting from reduced RBC development. Regulation of systemic iron levels represents an important first-line defense against infection. However, the role of iron in the immune response does not stop there. In the next section we will describe the essential, and often specific, role iron plays in several immune cell subtypes.

## Neutrophils and Iron

Neutrophils are the most numerous white blood cell in the body and are often the “first-responders” at the scene of invading pathogens or tissue damage. Neutrophils, as noted above, secrete lipocalin-2 to interfere with bacteria scavenging host iron from the bloodstream (Flo et al., 2004; Nairz et al., 2015). However, their primary function in host defense when they encounter microbes, is to ingest them into phagosomes, undergo a rapid burst of oxygen consumption and release anti-microbial proteins from granules into the phagosome to kill the microbes. The enzyme complex responsible for the oxygen burst is the NADPH oxidase (NOX)-2 complex at the plasma or phagolysomal membranes which channels electrons from NADPH in the cytosol to oxygen, generating superoxide (O_2_^**−**^) radicals within the phagosome (Vazquez-Torres et al., 2000). Superoxide radicals can free up Fe^2+^ iron and this can further react with hydrogen peroxide (H_2_O_2_) to give rise to the hydroxyl radicals (OH•) (Koskenkorva-Frank et al., 2013). All of these components - H_2_O_2_, O_2_^**−**^, and OH•–kill ingested microbes. Additionally, neutrophils have another iron-dependent metalloprotein in its arsenal; an enzyme called myeloperoxidase (MPO) contained in intracellular granules—which represents about 5% of the total protein within a neutrophil and is the “green protein” that gives pus and phlegm their green tinge. MPO is a hemoprotein and its Fe^3+^/Fe^2+^ redox states are critical to its role in producing anti-microbial effects (Arnhold et al., 2003). Chloride is also available in the phagosome through direct endocytosis or via chloride channel import such as the CFTR (cystic fibrosis transmembrane conductance regulator) channel (Di et al., 2006). MPO catalyzes the H_2_O_2_-mediated oxidation of chloride to form hypochlorous acid (HOCl) (Everse et al., 1991). Moreover, MPO oxidizes tyrosine to the tyrosyl radical again using H_2_O_2_ as an oxidizing agent (Everse et al., 1991). Both HOCl and tyrosyl radicals kill phagosome-restrained pathogens.

Other related hemoproteins include salivary peroxidase and lactoperoxidase (LPO) which are present in multiple human exocrine secretions, including tears, milk, saliva, and vaginal fluid, and also serves anti-microbial killing actions similar to MPO (Sarr et al., 2018). In addition, neutrophils produce large amounts of calprotectin and lactoferrin, both of which scavenge iron, thereby inhibiting bacterial proliferation (Nakashige et al., 2015). In addition to its role in scavenging iron, lactoferrin, through binding to its cognate receptor, promotes the maturation, migration and cell proliferation of many immune cells including macrophages and monocytes which impact on the efficacy of host responses in the course of infections (Actor et al., 2009; Legrand, 2012; Lepanto et al., 2019). Interestingly, eosinophils have a homolog of MPO called eosinophil peroxidase (EPX) which shares ~70% similarity at the amino acid level with neutrophilic MPO (Ten et al., 1989). However, unlike neutrophils which release MPO into the phagosome, eosinophils release their EPX-containing granules extracellularly to kill parasites to which they are attached (Acharya and Ackerman, 2014).

## Macrophages and Iron

Macrophages are another immune cell of the innate immune system. They are phagocytic cells which engulfs dead cells, pathogens as well as cancer cells. Moreover, they also present foreign antigens to the adaptive immune system (T and B cells) to activate that arm of our defense. However, macrophages also have an essential homeostasis role in our bodies in promoting RBC maturation as well as scavenging toxic free heme and hemoglobin from dying RBCs. Hemoglobin-containing RBC development in the bone marrow requires large amounts of iron (Figure 3). Macrophages and Kupffer cells scavenge heme from damaged or aged RBCs in the bone marrow, liver and spleen, and extract the Fe^2+^ iron using HO-1 and then either exports it back into the circulation through FPN or stores it in ferritin intracellularly (Knutson et al., 2005; Theurl et al., 2016). Interestingly, heme promotes monocyte differentiation into iron-recycling macrophages by inducing the master transcription factor for macrophage differentiation SPI-C, through direction inhibition of its repressor BACH1 (Haldar et al., 2014). This process is blocked by the action of hepcidin during times of infection to prevent iron release in the circulation, which would otherwise fuel extracellular pathogen growth (Ganz and Nemeth, 2015). In the case of infection with intracellular pathogens such a strategy would be counterproductive. Thus, macrophages infected with intracellular bacteria, induce a reprogramming of iron metabolism to limit intracellular iron availability for these pathogens. In mice, this is done by increased production of nitric oxide (NO) (Nairz et al., 2013), which activates the stress-responsive transcription factor, NRF2 resulting in transcriptional induction of FPN expression leading to subsequent increase of iron export from the cell, thus again restricting iron availability to the intracellular pathogen. Macrophages also express a phagolysosomal protein, known as NRAMP1 (natural resistance associated macrophage protein 1, SLC11A1) which confers resistance to intracellular microbes such as *Salmonella, Mycobacteria*, or *Leishmania* (Forbes and Gros, 2001). NRAMP1 acts as a transporter for divalent metals, including iron, and promotes iron export from the phagolyosome and then from the cytoplasm by induction of FPN expression (Nairz et al., 2007; Lim et al., 2018).

The intracellular iron equilibrium can regulate the polarization of macrophages into M1 (pro-inflammatory) and M2 (anti-inflammatory, pro-tissue healing) macrophages depending on their micro-environmental niche and local metabolic cues (Recalcati et al., 2012). *In vitro* M2-polarized macrophages present an iron-release prone phenotype, with higher FPN and lower ferritin expression levels than classically activated M1 macrophages. This allows the M2 macrophages to promote iron recirculation and aid in tissue healing, a process which requires large amount of iron to rebuild damaged tissue (Recalcati et al., 2010a). In contrast, M1 macrophages enhance iron uptake and storage, and display an attenuated iron-release phenotype favoring intracellular iron sequestration and storage, which has been shown to enhance antimicrobial effector functions such as increased pro-inflammatory TNFα expression while suppressing expression of the anti-inflammatory cytokine, IL-10 (Weiss et al., 1994; Mulero et al., 2002; Fritsche et al., 2008). Recently, several studies have investigated the iron-dependent immunometabolic switches involved in the activation of human macrophages. Acute deprivation of iron levels in human macrophages was shown to reprogram their transcriptional and metabolic responses, enhancing glycolytic-regulated genes via ATF4 while concurrently blocking mitochondrial oxidative phosphorylation (via reduced activity of the Fe-S containing respiratory chain enzymes NDUFS6 and SDHB), ultimately resulting in impaired cell proliferation and reduced severity in a macrophage-dependent mouse kidney autoimmune model (Pereira et al., 2019). Interestingly, a non-catalytic role for iron in human macrophage activation was demonstrated by which the trace metal is essential for modulating the nuclear membrane-binding ability of 5-LOX (5-lipoxygenase) (Dufrusine et al., 2019). 5-LOX is an enzyme important in the production of proinflammatory leukotrienes (LT). Upon macrophage stimulation, both cytosolic and nuclear 5-LOXs incorporate ferric iron (Fe^3+^) enabling its translocation to the nuclear envelope for LT production (Dufrusine et al., 2019). Therefore, iron ion binding represents an immediate post-translational mechanism not just supporting catalytic activities, as we have seen but also directly influencing intracellular trafficking and localization of proteins.

In addition, macrophage-dependent, and indeed microglia-dependent, regulation of iron homeostasis in the brain has been recently implicated in the maintenance of neuroinflammatory diseases such as multiple sclerosis. Myelin breakdown and the phagocytosis of its debris, including iron, occurs at active multiple sclerosis lesions. However, degeneration of cells in the brain also result in the deposition of iron in the extracellular space, which then induces waves of oxidative stress (via Fenton chemistry) in these brain regions (Craelius et al., 1982; Bagnato et al., 2011). Such microglial dystrophy has been observed in Alzheimer's brains as well as aged brains (Lopes et al., 2008).

Cancer immunotherapy, the ability to coax one's own immune system to seek and destroy cancer cells, has revolutionized anti-cancer therapies. Classical approaches of cancer immunotherapy have included inhibition of cell cycle checkpoints in T cells or blocking tumor-derived immune suppression strategies. Targeting iron homeostasis in immune cells, and in particular macrophages, has received recent interest. However, in the context of the tumor microenvironment (TME), the situation is quite complicated as M2 TAMs (tumor-infiltrating macrophages) which are “iron-releasing” will support tumor growth while M1 “iron-retaining” limit tumor progression (Jung et al., 2019). Therefore, novel approaches to manipulate iron homeostasis and metabolically re-program macrophages in the TME are currently being investigated. Iron-loaded macrophages display a pro-inflammatory M1-like phenotype which although can be associated with tissue damage, may also be used to exhibit anti-cancer responses. In lung cancer for example, iron-laden TAMs have been demonstrated to enhance production of ROS and pro-inflammatory cytokines (TNFα and IL-6) and to directly kill tumor cells (da Silva et al., 2017), prompting the exciting possibility of delivering iron to TAMs as a simple adjuvant therapeutic strategy. Indeed, iron oxide nanoparticles have been shown to modulate the IRF-5 (interferon regulatory factor 5) signaling pathway to enhance anti-tumor M1 macrophage polarization while at the same time down-regulate M2-assoicated arginase-1 (Gu et al., 2019). These studies provide new understandings of the role iron regulation plays in macrophage function and paves the way for designing advanced iron-based anti-inflammatory as well as anti-cancer technologies.

## T Cells and Iron

T cells, together with antibody-producing B cells, are the main components of the adaptive immune system. T cells can be cytotoxic (CD8^+^ T cells) as well as helper (CD4^+^ T cells) immune cells. CD8^+^ T cells can directly kill infected or cancer cells by releasing cytotoxic mediators such as perforin, while CD4^+^ T cells orchestrate and coordinate B cells and innate immune cells through release of various cytokines and chemokines. Proliferation and effector functions of T cells are energy expensive processes that require iron for the many metabolic and redox reactions involved as well as heme- and Fe-S-containing enzymes that are indispensable for cell division and cytokine production (Cronin and Penninger, 2007; Figure 3). T cells undergo rapid cellular expansion during development in the thymus as well as during an immune response, such as infection or cancer. Therefore, it is not surprising that one of the earliest cues for T cell activation and proliferation is the upregulation of the transferrin receptor (TFR, also called CD71) on the surface of T cells (Batista et al., 2004). Indeed within a minute of T cell receptor (TCR) engagement, surface TFR is upregulated from endosomal compartments and is localized to the immunological synapse (IS), the receptor-filled contact area between an antigen-presenting cell and a T cell, necessary for full T cell activation (Batista et al., 2004). TFR has also been hinted to play a role in T cell activation independent of its role in iron uptake as it also contributes to the signal transduction of TCR stimulation by the direct interaction of the ζ-chains of the CD3 co-receptor (Salmeron et al., 1995).

CD4^+^ helper T cells, when activated, can differentiate broadly into two major subtypes, T_H_1 (which coordinate macrophages and cytotoxic CD8^+^ T cell responses) and T_H_2 cells (which coordinate eosinophils, basophils, mast cells, and B cells). Inhibition of TFR affects the proliferation of T cell subtypes differently with T_H_1 immune responses being more sensitive to intracellular iron depletion than T_H_2 (Thorson et al., 1991; Mencacci et al., 1997). Moreover, it has been suggested that induction of T cell anergy, a process whereby an activated T cell becomes tolerant and functionally inactive (a desirable effect in cases of hyper T cell activation under autoimmune conditions and undesirable in the tumor microenvironment) involves TFR downregulation (Zheng et al., 2007). It has been also demonstrated using TFR blocking antibodies, that TFR-mediated iron uptake is essential for lymphocyte development and proliferation (Neckers and Cossman, 1983; Ned et al., 2003). Importantly, a mutation in the human *TFRC* gene, which codes for TFR, was identified in patients with combined immunodeficiencies (CID) (Jabara et al., 2015). Patients suffering from CID are highly susceptible to life-threatening infections due to a greatly diminished immune response mediated by the adaptive arm of the immune system, namely in T- and B-cell function. The identified single amino acid substitution in TRF1 (Y20H) affects iron uptake in both T and B cells resulting in defective development, proliferation and antibody type class switching (Jabara et al., 2015). Furthermore, hematopoietic-specific deletion of the ferritin heavy chain (*Fth*) gene results in reduced numbers of lymphocytes while other cell types such as granulocytes and monocytes are unaffected, again highlighting the essential nature of iron regulation to the adaptive immune system (Vanoaica et al., 2014). Ablation of *Fth* leads to an increase in the labile iron pool (LIP) within the cell contributing to increased oxidative stress and cell death, though when cells are stimulated the increased availability of Fe^2+^ iron enhances proliferative capacity before ultimately undergoing cell death, possibly due to proliferating cells using up the excess iron (Vanoaica et al., 2014). If intracellular iron stores could somehow be replenished, it would be interesting to then see whether enhanced proliferation would continue.

As mentioned above, hemolysis (RBC rupture) causes a massive release of heme-containing proteins which, when oxidized, liberate the heme/Fe moiety and thereby cause pro-oxidant, cytotoxic effects. Heme scavenging proteins such as hemopexin can bind free heme, thus clearing it from the circulation and delivering it to cells (Smith et al., 1988). Once internalized the heme is catabolized by HO-1 to produce iron as well as the cytoprotective molecules biliverdin and carbon monoxide (CO) (Tenhunen et al., 1969; Maines, 1988). CO has been shown to inhibit presentation of exogenous soluble antigens to CD8^+^ and CD4^+^ naïve T cells by blocking normal antigen trafficking in LPS-treated DCs (Tardif et al., 2013), whereas biliverdin induces tolerance to cardiac allografts by interfering with activation of nuclear factor of activated T cells (NFAT) and nuclear factor-κB (NF-κB), two transcription factors involved in interleukin-2 (IL-2) transcription and T cell activation and expansion (Yamashita et al., 2004). Thus, HO-1 exerts cytoprotective effects by reducing the pro-oxidative activity of heme and by re-programming metabolic traits, thereby limiting tissue damage in the course of inflammatory processes, including sepsis (Gozzelino et al., 2010; Weis et al., 2017). Accordingly, HO-1 deficient mice show enhanced CD4^+^ T cell activation (Poss and Tonegawa, 1997).

Patients with iron-overload in beta-thalassemia major have decreased CD4^+^ T cells but increased CD8^+^ T cell numbers (Porto and De Sousa, 2007), while patients suffering from hereditary hemochromatosis (HH) have apparently normal CD4^+^ and reduced CD8^+^ numbers, with the latter displaying a more “effector” phenotype, suggesting again that increased intracellular iron availability may have hyper-proliferative effects in T cells, although this may well**-**depend on which oxidation form of Fe is available (Costa et al., 2015). Further corroborating the link between intracellular iron stores and proliferation, cancer cells are known to stockpile intracellular iron through deregulating iron homeostasis mechanisms (Torti and Torti, 2013). Intriguingly, a significant association between almost 50% of genes involved in iron metabolism and breast cancer prognosis has been observed (Miller et al., 2011).

Iron deposition has long been known as a hallmark of many autoimmune diseases including in the brains of patients with neuroinflammatory diseases such as multiple sclerosis (MS), a brain inflammatory disease whereby autoreactive T cells facilitate CNS inflammation by secreting a variety of proinflammatory cytokines. Indeed, iron deprivation reduced EAE, a T cell dependent autoimmune mouse model of MS (Grant et al., 2003) and importantly, showed some beneficial improvements in MS patients (Hametner et al., 2013; Weigel et al., 2014). One recent mechanism of how iron drives T cell-mediated pathogenicity in MS centered on the regulation of GMCSF (granulocyte-macrophage colony-stimulating factor) by iron levels. GM-CSF is essential for the development and progression of EAE. Mice deficient in GM-CSF are resistant to EAE induction, and blockade of GM-CSF in wild-type mice suppresses ongoing disease (McQualter et al., 2001; Codarri et al., 2011). It was demonstrated that iron protects PCBP1 (Poly(RC)-binding protein 1), an RNA binding protein which stabilizes GMCSF mRNA, from caspase-mediated proteolysis thus leading to enhanced GMCSF production when iron levels are high within the T cell to drive inflammation (Wang et al., 2018). Moreover, increased intracellular bioavailable iron in CD4^+^ T cells has also been linked to the pathophysiology of systemic lupus erythematosus (SLE). Levels of intracellular iron were increased significantly in SLE CD4^+^ T cells compared to healthy controls and the researchers suggest a link between iron homeostasis and global DNA methylation status (Zhao et al., 2018), by which SLE T cells exhibit reduced DNA demethylation resulting in enhanced immune-related gene expression. Interestingly a link between intracellular iron levels and epigenetic programming has also been described to control B cell activation, proliferation and antibody responses at the level of H3K9 demethylation at the promoter region of the cell-cycle regulator, cyclin E1 (Jiang et al., 2019). Iron deficiency in mice led to dramatically attenuated antigen-specific antibody responses (Jiang et al., 2019). Intriguingly, human patients with iron deficiency also show significantly weakened antibody responses when challenged with the measles vaccine. Inhibited H3K9me demethylation at the promoter region of cyclin E1 (Jiang et al., 2019). These findings are quite significant in the light of the recent spate of measles and varicella outbreaks in areas with high vaccination coverage. It may therefore be interesting to further investigate the link between individual iron intake as well as iron serum levels and their ability to elicit adequate antibody production upon vaccination to reduce the risk of infection and disease.

Ferroptosis is an iron-dependent oxidative form of cell death associated with increased lipid peroxidation (Xie et al., 2016). It is morphologically and mechanistically distinct from apoptotic, necroptotic, and autophagic cell death (Xie et al., 2016). Moreover, ferroptosis can be blocked with iron chelators but not with inhibitors of apoptosis and necroptosis (Xie et al., 2016). The term ferroptosis was introduced to describe cell death induced by the compound erastin, which causes glutathione depletion through the glutamate-cystine antiporter system ${\text{X}}_{\text{c}}^{-}$ inhibition and consequently glutathione peroxidase 4 (GPX4) inactivation leading to accumulation of lipid peroxides (Ursini et al., 1982; Dixon et al., 2012). GPX4 is essential for the survival and expansion of recently activated T cells, indicating the importance of preventing lipid peroxidation and of iron dysregulation resulting in ferroptosis, in T cell proliferation (Matsushita et al., 2015). Iron is essential for the process of ferroptosis as free divalent iron (Fe^2+^) converts the hydrogen peroxide (H_2_O_2_) produced by mitochondrial respiration into the toxic free hydroxyl radical (OH•) through Fenton chemistry which then “takes” electrons from lipids resulting in lipid peroxidation and ferroptosis. As mentioned above, tumors have developed mechanisms to suppress T cells in the tumor microenvironment (TME) and that the field of cancer immunotherapy focuses on re-establishing cytotoxic CD8^+^ T cell-mediated killing of cancer cells by, for example, the use of checkpoint inhibitors. Notably, it has recently been demonstrated that restored immunotherapy-activated CD8^+^ cytotoxic T cells downregulate the expression of the system ${\text{X}}_{\text{c}}^{-}$ on tumor cells through IFNγ leading to enhanced lipid peroxidation and ferroptosis of tumor cells (Wang et al., 2019), suggesting a potential combinatorial therapeutic approach of removing checkpoints as well as targeting iron metabolism, for more effective cancer immunotherapy.

## Tetrahydrobiopterin (BH4)—Linking Iron Homeostasis to Mitochondrial Function in Activated T Cells

**Figure 4:**
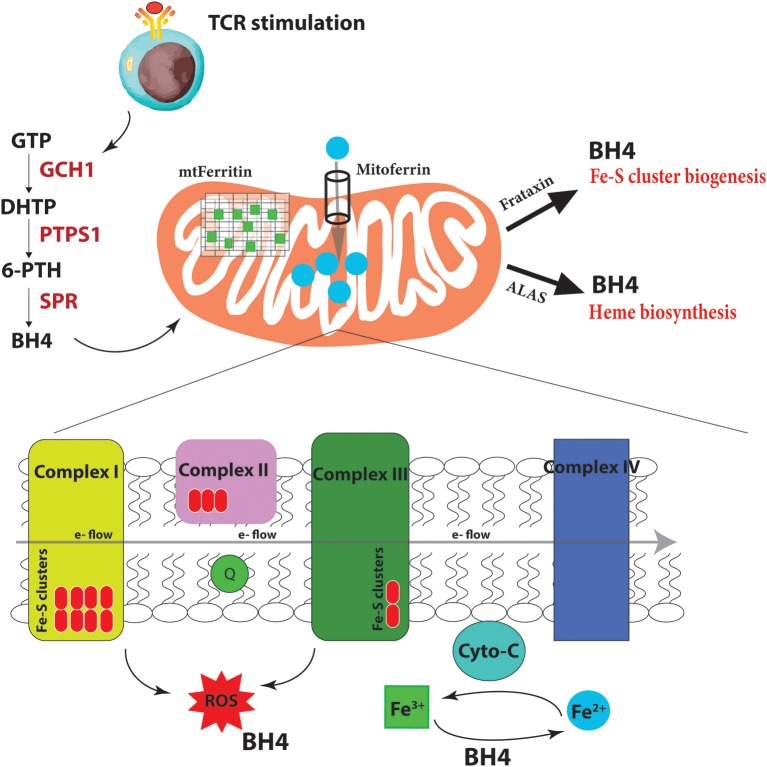
BH4 enhances iron-dependent mechanisms upon T cell activation. Upon T cell receptor (TCR) stimulation, T cells alter their metabolic programming, such as promoting energy-producing mitochondria and electron transport chain (ETC) activity, to be able to proliferate and carry out effector functions. These increased metabolic needs require additional regulators for efficient ETC function to limit dangerous ROS byproducts due to the increased energy demands. To accomplish this, T cells induce the expression of the enzyme GCH1 which produces the metabolite BH4 after stimulation. BH4 can not only act as a superoxide ROS scavenger but also directly reduce Fe^3+^ to Fe^2+^ and therefore affect cytochrome C (cyto-C) activity in the ETC. Other iron-regulated processes such as heme and Fe-S biogenesis, as well as the function of various iron-dependent metalloproteins, may also be affected. Under BH4 deficiency, T cells show dysfunctional ETC, enhanced ROS, and reduced ATP production in activated T cells. Moreover, the T cells show dysregulated Mitoferrin, Frataxin, HO-1, Ferritin and overall reduced iron levels in the cell suggesting that BH4 deficiency affects iron metabolism and the cell compensates by trying to increase Fe^2+^ levels in the mitochondria. GTP, guanosine triphosphate; PTPS, 6-pyruvoyl tetrahydropterin synthase; SPR, sepiapterin reductase; DHTP, dihydroneopterin triphosphate; 6-PTH, 6-pyruvoyl tetrahydropterin; BH4, tetrahydrobiopterin; ROS, reactive oxygen radicals; ALAS, aminolevulinic acid synthase; e-, electron.

Iron homeostasis, its regulation through uptake, use, storage and export, influences the activity of metabolic pathways to couple the activation, growth and survival of T cells. Our laboratory has recently uncovered a novel role for a metabolite, linking iron regulation to T cell effector function (Cronin et al., 2018). Tetrahydrobiopterin, known as BH4, has been till now almost exclusively studied as an essential co-factor for several enzymes with critical physiologic and metabolic functions, including the three nitric oxide synthases (neuronal, inducible and endothelial NOS), alkylglycerol mono-oxygenase (AGMO), and aromatic amino acid hydroxylases (phenylalanine, tryptophan and tyrosine hydroxylases) (Werner et al., 2011). Through these enzymes, BH4 is required for nitric oxide (NO) production, metabolism of ether lipids, phenylalanine catabolism, and synthesis of the amine neurotransmitters norepinephrine, epinephrine, serotonin, and dopamine. GTP cyclohydrolase I (GCH1) is the rate-limiting enzyme for BH4 biosynthesis (Werner et al., 2011; Figure 4). In terms of the immune system, BH4-dependent nitric oxide (NO) production by inducible nitric oxide synthase (iNOS) in macrophages is compromised in *Gch1*-deficient macrophages (McNeill et al., 2015) although these cells have an enhanced ability to control the growth of *Mycobacterium tuberculosis* through NO- and BH4-independent mechanisms, which are enhanced upon BH4 deficiency (McNeill et al., 2018).

We and others have shown that GCH1 is expressed in T cells but only after TCR stimulation (Chen et al., 2011; Cronin et al., 2018). Using multiple T cell specific ablation strategies, we demonstrated that genetic inactivation of GCH1 or pharmacological inhibition of the terminal enzyme in the biosynthetic pathway of BH4, sepiapterin reductase (SPR), results in severely impaired proliferation of T cells (Cronin et al., 2018). In various models of T cell development autoimmunity or asthma, BH4 blockage significantly reduces T cell effector function and infiltration into affected tissues. However, interestingly, T cell development or homeostasis as well as B cell and regulatory T cell (T_reg_) functions are unaffected by inhibition of this pathway. Furthermore, by enhancing BH4 production, either genetically or pharmacologically, we observed an opposing phenotype, where the T cells are hyper-activated, displaying stronger effector functions. In orthotopic cancer transfer models, BH4-overproducing T cells have increased anti-cancer immunity, resulting in greatly reduced cancer burden (Cronin et al., 2018).

What is BH4 doing in these cells? In genetic inactivation models, we did not detect any differences in iNOS expression or NO production in T cells, nor any detectable signs of activated T cells producing biogenic amine neurotransmitters that require co-factor BH4. Gene expression profiling comparing activated wild-type T cells to those lacking GCH1 (and hence displaying BH4 deficiency) pointed instead to iron regulation. We indeed observed increases at the protein level of Mitoferrin, the mitochondrial Fe^2+^ importer; HO-1, which breaks heme into Fe^2+^; ferritin, the storage regulator of Fe^3+^, as well as frataxin, responsible for Fe-S biogenesis. Moreover, we confirmed data from almost 50 years ago that BH4 alone, in a co-factor independent manner, can reduce cytochrome-C-Fe^3+^ to cytochrome-C-Fe^2+^ at physiological concentrations and efficacy (Archer et al., 1972; Figure 4). These data suggested that when T cells become stimulated through TCR engagement, the increased metabolic needs of the dividing, cytokine-producing effector cell requires enhanced Fe^2+^-dependent mechanisms for, among many others, efficient electron transfer chain (ETC) during mitochondrial respiration and increased ATP synthesis. Under conditions of BH4 deficiency, as in our *Gch1*-ablated T cells, cytochrome-C reduction is compromised, resulting in dysfunctional mitochondrial respiration, increased superoxide formation and, ultimately diminished ATP production, all of which we observed (Cronin et al., 2018). We could rescue the mitochondrial respiration defect by supplying reduced cytochrome-C-Fe^2+^ directly to the mitochondria of *Gch1*-ablated T cells after stimulation.

Fe^2+^ is not only needed for metalloproteins like cytochrome-C to function optimally but also for Fe-S cluster biogenesis, which are essential for complex I and II activity of the ETC, and heme production (Figure 4). A common phenotype of defective Fe-S biogenesis is mitochondrial iron loading, resulting in mitochondrial dysfunction and oxidative stress (Reeve et al., 2012). Indeed, such Fe-S mitochondrial disruptions can cause cytosolic iron depletion (Huang et al., 2011). We observed an overall depletion of total iron in T cells after activation under BH4-deficient conditions. A general decrease in Fe^3+^ reduction resulting in a scarcity of Fe^2+^ and increase of Fe^3+^ would affect many aspects of cellular metabolism (Volani et al., 2017) which contribute to the reduced T cell proliferation observed with BH4-deficiency. By increasing the levels of mitoferrin, HO-1, and frataxin, the BH4-deficent T cells attempt to increase the import of Fe^2+^ into the mitochondria for Fe-S biogenesis and other iron-dependent processes. Interestingly, we also observed enhanced secretion of IFNγ in *Gch1*-deficient T cells following antigen receptor-mediated activation. IFNγ has a strong regulatory role in iron homeostasis as it enhances the expression of cellular iron-regulating genes such as HO-1 but also reduces the iron-exporting gene ferroportin (Ludwiczek et al., 2003; Nairz et al., 2015). This may explain the enhanced IFNγ observed in *Gch1*-deficient T cells, to increase the amount of Fe^2+^ by preventing its export (through FPN downregulation) while increasing HO-1 to provide more bioavailable Fe^2+^ from heme degradation for the mitochondria. Alternatively this may be a compensatory mechanism of the cell responding to low BH4 levels, as IFNγ can also upregulate GCH1 in macrophages (Oexle et al., 2003).

## Therapeutic Targeting of the BH4 Pathway to Control Autoimmunity and Cancer

Intriguingly, sulfasalazine, a drug used for decades to treat autoimmune inflammatory bowel disease (IBD) and psoriatic arthritis (Gupta et al., 1990), is now recognized as targeting the BH4 pathway by inhibiting SPR (Haruki et al., 2013), in addition to other targets, such as NFκB (Wahl et al., 1998). Interestingly, high doses of sulfasalazine are associated with RBC abnormalities (Pounder et al., 1975). A more specific and potent target for reducing activity in the BH4 synthetic pathway could therefore provide new opportunities to treat autoimmune diseases. To this end, we developed a novel BH4 inhibitor, QM385, which targets SPR, and blocks both mouse and human T cell proliferation at nanomolar potency with good oral bioavailability and no observable side-effects at immune suppressant exposures (Cronin et al., 2018). Importantly, in several different models of T cell-mediated autoimmunity, such as intestinal inflammation, experimental allergic encephalitis (EAE), psoriasis, and T cell-driven asthma, the genetic or pharmacologic inhibition of the BH4 pathway markedly abrogated the severity of autoimmunity. We also demonstrated that human peripheral blood mononuclear cells (PBMCs) when stimulated through the T cell receptor (TCR) produce BH4 and that QM385-mediated inhibition of BH4 diminished the proliferative capacity of healthy human donor T cells (Cronin et al., 2018). Our work therefore provides strong evidence that targeting this pathway offers a novel therapeutic avenue for the treatment of autoimmunity.

We also showed that by enhancing BH4 genetically, or pharmacologically with BH4 itself, T cells displayed heightened proliferation and, importantly, increased anti-tumor activity (Cronin et al., 2018). We observed that with increased BH4, mitochondrial respiration and ATP production were also amplified, however, we did not see a surge in total intracellular iron contents. Whether the ratio of Fe^3+^ to Fe^2+^ or the amount of metabolically active iron is affected needs to be further investigated. As discussed above, T cells from patients suffering from HH and iron overload exhibit abnormal proliferation, differentiation and functionality (Walker and Walker, 2000). Hence, the T cell proliferative effect of increased BH4 may very well-affect iron regulation. Whether manipulations of the pathway will ameliorate T cell function and immune responses both in general and in situations of pathologic iron accumulation needs to be tested in future experiments.

## Conclusions

In this review we have highlighted the major pathways and immune cells involved in iron regulation, from initial uptake in the gut to the utilization of iron for Fe-S clusters, heme biogenesis and mitochondrial function. Nature has evolved complex regulatory pathways to control iron levels, not only in the circulation, but within cells. Invading pathogens can hijack these iron regulatory networks for their own benefit and the hosts have developed responses to counter such iron scavenging pathogens. Different immune cells use iron for different effector functions ranging from “respiratory bursts” by neutrophils, killing intracellular pathogens for macrophages, or for T cells, regulating iron metabolism required for efficient proliferation. Competition for iron at the host-pathogen interface impacts the course of infection and alterations of iron homeostasis in inflammation can impair erythropoiesis resulting in anemia. With recent technological advances and whole transcriptomic, proteomic and metabolic profiling, exciting new insights into how immune cells regulate, and are regulated by iron have emerged. We identified BH4 as a metabolite induced in antigen receptor-stimulated T cells. In such activated T cells, BH4 regulates both the reduction of Fe^3+^ and increases the availability of Fe^2+^ in the cell, which aid activated T cells in coordinating the increased metabolic needs for effector functions. Targeting this pathway offers a novel therapeutic and new mode-of-action opportunity to control T cell proliferation, and in this way help to control autoimmunity or enhance anti-cancer immunity.

## Author Contributions

SC, GW, CW, and JP wrote and edited the review.

### Conflict of Interest

The authors declare that the research was conducted in the absence of any commercial or financial relationships that could be construed as a potential conflict of interest.
